# Editorial: Innate immunity and cross-talk with microflora in the regulation of immune recognition and polarization during immune-related diseases

**DOI:** 10.3389/fimmu.2023.1335238

**Published:** 2023-11-20

**Authors:** Haitao Zhu, Zhongwei Liu

**Affiliations:** ^1^ Department of Pediatrics (No. 3 Ward), Northwest Women’s and Children’s Hospital, Xi’an, Shaanxi, China; ^2^ Allergy & Immunology Clinic, Northwest Women’s and Children’s Hospital, Xi’an, China; ^3^ Department of Cardiology, Shaanxi Provincial People’s Hospital, Xi’an, Shaanxi, China

**Keywords:** innate immunity, microbiota, cross talk, immune recognition, immune polarization

Recent years have witnessed a surge in research exploring the interactions between microflora (microbiota) and innate immunity. The human body, densely populated by a diverse array of bacteria, serves as a nexus where microbial inhabitants and host organisms co-evolve. This symbiosis gives rise to a multifunctional microbiota ecosystem integral to both physiological and pathological processes in humans. These processes encompass a broad spectrum, from nutrient synthesis and metabolic functions to key roles in digestive processes, immune regulation, and systemic defense mechanisms.

At the heart of this interaction lies the innate immune system, serving as a critical interface between the host and its microbiota. Commensal bacteria play a pivotal role in shaping the structural development of gut-associated lymphoid tissues (GALTs), priming immune cells for an efficacious response, and influencing adaptive immunity. Thus, the microbiota-innate immunity axis is instrumental in sustaining immune homeostasis and orchestrating immune cell polarization in response to various stimuli.

Unraveling the intricacies of this interplay, and the mechanisms underpinning it, holds immense potential for advancing our understanding of immune-related pathologies, including atopic disease (AD), autoimmune diseases (AID), metabolic disorders, infectious diseases, and cancers.

This Research Topic is dedicated to probing the roles and underlying mechanisms of microbiota, bacterial components, and metabolites in maintaining immune homeostasis and influencing disease progression. Our objective is to deepen our understanding of the immunological interactions with microbiota in the context of immune-related disorders and to explore innovative strategies for modulating the microbiota-innate immunity axis to mitigate the prevalence of immune diseases.

Ankylosing spondylitis (AS), a chronic autoimmune disease marked by inflammation of the spine and sacroiliac joints, is explored in depth by Song et al. Their review, “*Role of the Microbiome and its Metabolites in Ankylosing Spondylitis*,” delves into the contribution of the gut microbiota (GM) to AS pathogenesis, highlighting potential therapeutic targets. The authors elaborate on various mechanisms, including the interaction between gut microbiota and HLA-B27, increased intestinal epithelial permeability, gut mucosa immune imbalance, impaired gut barrier function, and disrupted bone metabolism. Their findings suggest that microbial dysbiosis may activate intestinal inflammation, triggering immune responses in AS.

Jalandra et al., in “*Inflammatory and Deleterious Role of Gut Microbiota-Derived Trimethylamine on Colon Cells*,” examine the impact of trimethylamine (TMA), a microbial metabolite derived from dietary quaternary amines, on colonic epithelial cells and in mouse models. Their results indicate TMA’s genotoxic and cytotoxic effects, with animal studies demonstrating significant intestinal damage and inflammation.

Ou et al., in their review “*The Gut-Lung Axis in Influenza A: The Role of Gut Microbiota in Immune Balance*,” summarize alterations in GM observed in human and animal models during influenza A virus (IAV) infection. The authors propose that GM influences innate and adaptive immunity, potentially via toll-like receptors (TLRs), RIG-I-like receptors (RLRs), inflammasome assembly, and modulating CD4+ and CD8+ T cell functions. GM modulations may be prospective treatment options to promote anti-IAV immune homeostasis.

Wang et al., in “*Orally Administered Lactiplantibacillus plantarum OLL2712 Decreased Intestinal Permeability, Especially in the Ileum: Ingested Lactic Acid Bacteria Alleviated Obesity-Induced Inflammation by Collaborating with Gut Microbiota*,” demonstrate that *Lactiplantibacillus plantarum* OLL2712 can alleviate obesity-related gut microbiota imbalances in a mouse model. The authors report reduced intestinal inflammation and enhanced gut barrier function, attributed to the regulation of key gene expressions.

Wassie et al., in “*Microbiome-Metabolome Analysis Reveals Alterations in the Composition and Metabolism of Caecal Microbiota and Metabolites with Dietary Enteromorpha Polysaccharide and Yeast Glycoprotein in Chickens*,” show that dietary supplementation with *Enteromorpha polysaccharide* (EP) and *Yeast glycoprotein* (YG) modulates intestinal microbiota and their metabolites. This finding provides insight into potential feed additives and therapeutic approaches in metabolic diseases.

Zhang et al., in “*PRR-Mediated Immune Response and Intestinal Flora Profile in Soybean Meal-Induced Enteritis of Pearl Gentian Groupers*,” demonstrate significant correlations between intestinal flora variations and activation of pattern recognition receptors (PRRs) in soybean meal-induced enteritis in pearl gentian groupers. Their work may pave the way for understanding immune regulation and PRR mechanisms in preclinical models.

Abdelhamid et al., in “*Nlrp12 Deficiency Alters Gut Microbiota and Ameliorates Faslpr-Mediated Systemic Autoimmunity in Male Mice*,” reveal that Nlrp12 deficiency attenuates autoimmune responses and modifies gut microbiota in a sex-dependent manner, offering novel insights into NLRP12’s immunoregulatory role in systemic AIDs.

Lastly, Huang et al., in their bidirectional Mendelian randomization study “*Causal Relationships Between Gut Microbiota and Programmed Cell Death Protein 1/Programmed Cell Death-Ligand 1*” establish potential causal links between gut microbiomes and the immune checkpoint proteins PD-1/PD-L1, expanding the scope of immunotherapy and cancer treatment.

In conclusion, this Research Topic provides a platform for cutting-edge research exploring the dynamic interplay between innate immunity and microbiota in immune-related diseases, heralding novel therapeutic avenues.

## Author contributions

HZ: Writing – original draft, Writing – review & editing. ZL: Writing – original draft, Writing – review & editing.

